# Relationship between Pyochelin and *Pseudomonas* Quinolone Signal in *Pseudomonas aeruginosa*: A Direction for Future Research

**DOI:** 10.3390/ijms25168611

**Published:** 2024-08-07

**Authors:** Xin Ma, Jing Zeng, Wei Xiao, Wenwen Li, Juanli Cheng, Jinshui Lin

**Affiliations:** Shaanxi Key Laboratory of Chinese Jujube, College of Life Sciences, Yan’an University, Yan’an 716000, China; ma20230718xin@163.com (X.M.); 17868811824@163.com (J.Z.); 18792388186@163.com (W.X.); 15129750408@163.com (W.L.)

**Keywords:** *Pseudomonas aeruginosa*, quorum sensing, iron uptake, pyochelin, PQS, transporter

## Abstract

*Pseudomonas aeruginosa* is an opportunistic pathogen that requires iron to survive in the host; however, the host immune system limits the availability of iron. Pyochelin (PCH) is a major siderophore produced by *P. aeruginosa* during infection, which can help *P. aeruginosa* survive in an iron-restricted environment and cause infection. The infection activity of *P. aeruginosa* is regulated by the *Pseudomonas* quinolone signal (PQS) quorum-sensing system. The system uses 2-heptyl-3-hydroxy-4-quinolone (PQS) or its precursor, 2-heptyl-4-quinolone (HHQ), as the signal molecule. PQS can control specific life processes such as mediating quorum sensing, cytotoxicity, and iron acquisition. This review summarizes the biosynthesis of PCH and PQS, the shared transport system of PCH and PQS, and the regulatory relationship between PCH and PQS. The correlation between the PQS and PCH is emphasized to provide a new direction for future research.

## 1. Introduction

Iron is an essential element required for many cellular processes in all organisms and is a cofactor for many enzymes involved in key metabolic processes, such as cellular respiration, nucleotide biosynthesis, DNA replication, transcription, and repair [1,2]. Under aerobic conditions, iron exists in the form of Fe^3+^ [3,4]. Conversely, iron is abundant as Fe^2+^ in an anaerobic environment or at low pH [3,4]. To cope with its complex environment, *Pseudomonas aeruginosa* evolved different strategies to obtain iron, including: (1) the uptake of the host’s iron-carrier heme molecule; (2) the uptake of Fe^2+^ through the Feo system and phenazine; (3) absorption of siderophores produced by other microorganisms; and (4) production of Fe^3+^ chelating siderophores (pyoverdine and pyochelin, PCH) [3,5,6,7].

In addition to the two siderophores for obtaining Fe^3+^, *P. aeruginosa* can also secrete 2-heptyl-3-hydroxy-4-quinolone, known as the *Pseudomonas* quinolone signal (PQS), which can also chelate Fe^3+^ or mediate iron uptake [8,9,10]. PQS is a signaling molecule in the *pqs* quorum-sensing (QS) system of *P. aeruginosa*, which has been shown to not only affect gene transcription but also directly bind to some unrecognized protein receptor [11,12,13,14,15]. According to our previous research, PCH and PQS can enter *P. aeruginosa* through the same pathway [9,16]. This indicates that there is a correlation between PCH and PQS in some respects. In this review, by organizing relevant data (as of 2024), we describe the biosynthetic processes of PCH and PQS and discuss their relationship in biological processes. This is the first report on the relationship between PCH and PQS, which will provide theoretical guidance for understanding the physiological mechanisms of *P. aeruginosa*.

## 2. Biosynthesis of PCH and PQS

### 2.1. PCH Biosynthesis

**Figure 1:**
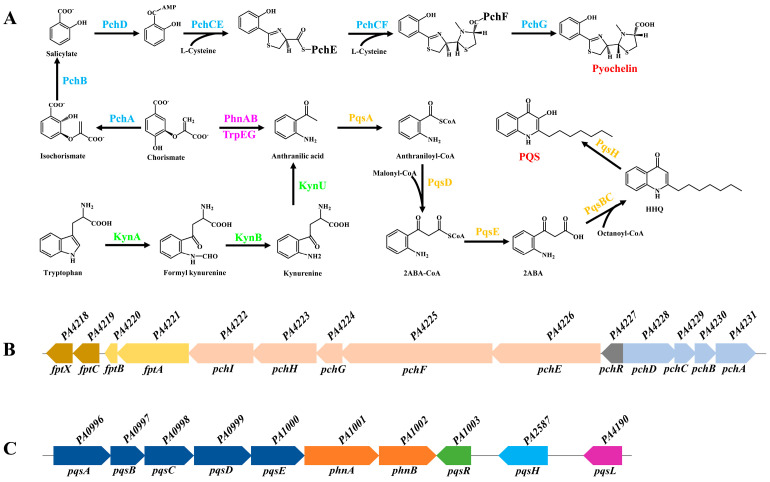
Biosynthetic pathway of pyochelin (PCH) and *Pseudomonas* quinolone signal (PQS) in *P. aeruginosa*. (**A**) Biosynthesis of PCH and PQS in *P. aeruginosa*. The black arrows indicate the direction of biosynthesis; the sky–blue font indicates the enzymes involved in PCH biosynthesis. The purple font indicates the enzymes involved in the conversion of chorismate to anthranilic acid. The green font indicates the enzymes involved in the kynurenine pathway, which converts tryptophan to anthranilic acid. The orange font indicates the enzymes involved in PQS biosynthesis. The red font shows the chemical structure of PCH and PQS. The detailed biosynthesis processes are described in the text. (**B**) PCH biosynthetic genes in *P. aeruginosa*. (**C**) PQS biosynthetic genes in *P. aeruginosa*. The square arrow indicates the direction of the gene transcription. Different colors represent different transcription units. *PA* gene numbers refer to the PAO1 sequence.

PCH is a non-ribosomal peptide that is a salicylate-based siderophore formed by the condensation of salicylate and two cysteines [5,6]; therefore, its biosynthesis requires the participation of non-ribosomal polypeptide synthases (NRPSs) [5,6,17]. NRPSs are a complex consisting of multiple modules [18]. Each module contains multiple domains and is responsible for adding an amino acid to the peptide chain [18]. These domains are generally an adenylation domain (A domain), peptidyl carrier protein (PCP domain), and condensation domain (C domain) [18]. In addition, the last module of NRPSs usually contains a thioesterase domain (T domain) that terminates the assembly of peptide chains [18]. Two operons are involved in PCH biosynthesis: *pchDCBA* and *pchEFGHI* [5]. The biosynthesis of PCH begins with PchA, an isochorismate synthase [17]. It can convert chorismate into isochorismate [19,20]. Second, isochorismate is converted to salicylate by PchB, an isochorismate pyruvate lyase [21,22,23]. Third, salicylate is activated by the salicylate-adenylating enzyme PchD and transferred to NRPS PchE [22,24,25,26]. Under the control of the thioesterase PchC, adenylated forms of salicylate bind to L-cysteine [5,17,21,27]. PchC can remove wrongly charged molecules from the NRPS PCP domain and does not replace the function of the NRPS T domain [21]. Subsequently, the intermediate hydroxyphenyl-thiazoline (HPT) is formed after epimerization and cyclization of salicylate and L-cysteine. HPT is released from PchE to give dihydroaeruginoique (DHA) [5,17,21]. Fourth, DHA binds to the second L-cysteine through NRPS PchF, a process that also requires the participation of PchC [17,21]. PchF contains a cyclization domain that can cyclize the second L-cysteine to form hydroxyphenylbis-thiazoline (HPTT) [17,22,24]. Finally, after HPTT is methylated by PchF and reduced by PchG, PCH is released by the thioesterase domain [5,17,22,28,29]. The biosynthetic pathway is shown in Figure 1.

### 2.2. Biosynthesis of PQS

Anthranilic acid is a precursor compound for the synthesis of PQS [30]. There are two main sources of this compound: chorismate and tryptophan [30,31,32]. Chorismate is converted to anthranilic acid under the action of anthranilate synthases TrpEG and PhnAB [32,33]. Palmer et al. suggested that the anthranilic acid produced by TrpEG is used for tryptophan biosynthesis, whereas that produced by PhnAB is used for quinolone signal molecular PQS biosynthesis [31,32,34]. The expression of these two enzymes varies with cell density [31]. TrpEG is mainly expressed at low density, whereas PhnAB is mainly expressed at high density [31]. However, in the presence of tryptophan, *phnAB* knockout mutants still produce PQS [31]. Tryptophan decomposes into anthranilic acid via the kynurenine pathway [30,35]. Tryptophan is first converted to formyl-kynurenine by tryptophan 2,3-dioxygenase KynA [30,36], and then formyl-kynurenine is converted to kynurenine by the kynurenine formamidase KynB [30,36]. Finally, the conversion of kynurenine to anthranilic acid is catalyzed by the kynureninase KynU [30,36].

The biosynthesis of PQS involves multiple genes in the *P. aeruginosa* genome, such as *pqsABCDE* and *pqsH* [37]. PQS biosynthesis begins with PqsA, an anthranilate-coenzyme A ligase that converts anthranilate to anthraniloyl-coenzyme A [38,39]. Second, 2-aminobenzoylacetyl-coenzyme A (2-ABA-CoA) is synthesized by anthraniloyl-coenzyme A and malonyl-coenzyme A under the control of the anthraniloyl transferase PqsD [40,41]. Third, 2-ABA-CoA is hydrolyzed by the thioesterase PqsE to produce 2-aminobenzoylacetate (2-ABA) [38,42]. Furthermore, a broad specificity thioesterase, TesB, can partially offset the function of PqsE [42]. Fourth, 2-ABA condenses with octanoyl-coenzyme A to form 2-heptyl-4-quinolone (HHQ) in the presence of the dimer PqsBC [41,42,43]. Finally, HHQ is hydroxylated by monooxygenase PqsH under aerobic conditions to form PQS [16,44]. PQS is a unique cell-to-cell signal, but the potential mechanism of PQS transport by *P. aeruginosa* to the extracellular environment remains unclear. The biosynthetic pathway is illustrated in Figure 1.

## 3. Shared Transport System

### 3.1. PCH and PQS Share Outer Membrane Transporter FptA to Mediate Iron Uptake

**Figure 2:**
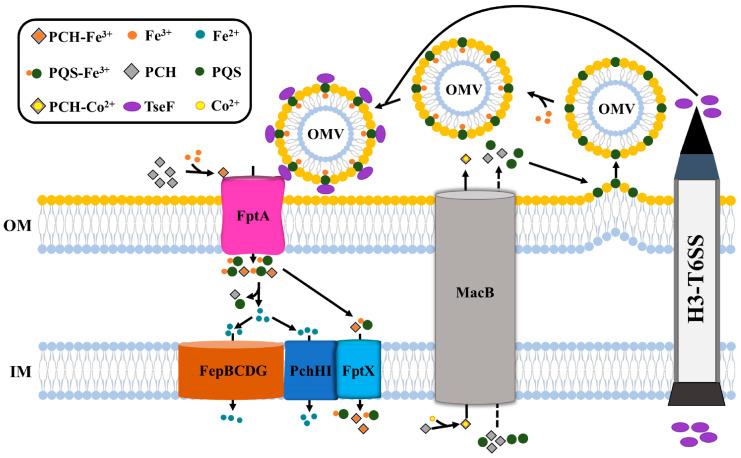
Proposed schematic representation of PCH and PQS transport in and out of *P. aeruginosa* cells. PQS bends the outer membrane to form outer membrane vesicles (OMVs) and chelates Fe^3+^ to form an OMV–PQS–Fe^3+^ complex. With the mediation of the T6SS effector TseF, PQS–Fe^3+^ on OMV is transported into the periplasm through the outer membrane receptor FptA. Similarly, PCH chelated with Fe^3+^ also enters the periplasm through FptA. In the periplasm, part of the complex of PQS–Fe^3+^ and PCH–Fe^3+^ enters the cytoplasm directly through FptX, whereas the other part of the complex of PQS–Fe^3+^ and PCH–Fe^3+^ dissociates through an unknown mechanism, and siderophore-free iron enters the cytoplasm through the inner membrane transporters PchHI and FepBCDG. MacB is a potential efflux pump that mediates the secretion of PCH (or the PCH–Co complex) and PQS.

PCH is a tetradentate ligand that can chelate Fe^3+^ via two amines and two alcohols [45]. Its stoichiometry is one ferric ion to two PCH molecules [45]. FptA is the only outer membrane transporter of PCH–Fe^3+^ assimilated by *P. aeruginosa* cells [45,46,47]. The crystallographic structure of FptA indicates that it is a typical TonB-dependent transporter, a transmembrane 22 β-stranded barrel occluded by an N-terminal domain (called the plug or cork domain) that contains a mixed four-stranded β-sheet [48,49,50,51]. In addition to transporting PCH–Fe^3+^, FptA also transports other PCH–metal complexes (PCH–Zn^2+^ or PCH–Ni^2+^) and acts as a receptor for pyocin S5 and some bacteriophages to enter cells [52,53]. Recently, our team found that FptA is also involved in the uptake of PQS–Fe^3+^ by *P. aeruginosa* [9,16]. This is a complex process involving FptA, TseF (a type VI secretion system effector), and PQS [9,16]. When PQS is secreted outside the cell, it can be embedded in the outer membrane, causing it to bend to form outer membrane vesicles (OMVs) [9,16]. Under iron-limited conditions, PQS in OMV forms an OMV–PQS–Fe^3+^ complex with extracellular Fe^3+^ [9,16], and its stoichiometry is one ferric ion to three PQS molecules [54]. Subsequently, the TseF protein can bind to PQS–Fe^3+^ on OMV [9,16]. The PQS–Fe^3+^ complex is then pulled to the outer membrane receptor FptA by TseF [9,16]. Finally, FptA transports PQS–Fe^3+^ into the cell [9,16]. In summary, PCH and PQS in *P. aeruginosa* share the outer membrane transporter FptA to mediate iron uptake (Figure 2).

### 3.2. PCH and PQS Share Inner Membrane Transporters FptX, PchHI, and FepBCDG to Mediate Iron Uptake

FptX is a known inner membrane transporter that mediates PCH–Fe^3+^ uptake [6]. It is a proton-motive-dependent permease that belongs to a new family of single-subunit siderophore transporters [16,46,55]. After being transported to cells by FptX, PCH–Fe^3+^ acts as a ligand for the transcription regulatory factor PchR, binds to PchR, and activates the expression of related genes (including the PCH biosynthetic operons *pchDCBA* and *pchEFGHI*, and the PCH–Fe^3+^ uptake operon *fptABCX*). These form an autoregulatory loop [16,55,56]. The transport efficiency of FptX for PCH–Fe^3+^ has been reported to be only approximately 50% [55], which means that other inner membrane transport channels are involved in this process [16]. Recently, we found that the inner membrane transporters, PchHI and FepBCDG, are associated with the uptake of PCH–Fe^3+^ and PQS–Fe^3+^ [16]. PchHI belongs to the ABC transporter family, which is encoded by the last two genes of the PCH synthesis operon *pchEFGHI* and can form heterodimers [56]. FepBCDG is an inner membrane transporter complex of the ABC family, which is composed of FepB (*PA4159*), FepC (*PA4158*), FepD (*PA4160*), and FepG (*PA4161*) [16]. However, unlike FptX, FepBCDG and PchHI do not participate in the autoregulatory loop involving PchR [16]. This is consistent with the conclusions of Roche B et al., where part of PCH–Fe^3+^ dissociates in the periplasm through an unknown mechanism, and the free iron is transported into the bacterial cytoplasm by PchHI [16,56]. Therefore, we speculated that both FepBCDG and PchHI may play a role in transporting siderophore-free iron to the cytoplasm [16]. In addition, we found that in iron-rich or iron-limited media, the exogenous addition of PQS–Fe^3+^ could activate the expression of *phzA1* (the pyocyanin synthesis gene) and *lecA* (the lectin gene) [16]. When the three inner membrane transporters (FptX, PchHI, and FepBCDG) were deleted, this effect disappeared. This indicates that the function of PQS–Fe^3+^-mediated QS regulation is dependent on FptX, PchHI, and FepBCDG. Interestingly, we also found that FptX, PchHI, and FepBCDG can interact with each other to form a larger complex that mediates the uptake of PCH–Fe^3+^ and PQS–Fe^3+^ [16].

In conclusion, during the transport process (Figure 2), once PCH–Fe^3+^ and PQS–Fe^3+^ enter the *P. aeruginosa* periplasm, they have two different fates. The first fraction of PCH–Fe^3+^ and PQS–Fe^3+^ is directly transported to the cytoplasm through FptX [16,45]. The second fraction of PCH–Fe^3+^ and PQS–Fe^3+^ dissociates into PCH, PQS, and free iron in the periplasm through unknown mechanisms, and free iron is further transported to the cytoplasm through the ABC transporters PchHI and FepBCDG [16,45].

### 3.3. Do PCH and PQS Share the Same Secretory Pathways?

As mentioned earlier, PCH and PQS in *P. aeruginosa* share membrane transporters that mediate iron uptake. It is easy to think that these two molecules may also share secretory pathways. However, the secretory pathway of the *P. aeruginosa* PQS remains unknown. As precursors of PQS, HHQ and PQS have similar chemical structures [38,57,58], which makes it possible that they have similar secretory modes. Efflux pumps are transporters on the bacterial membrane that regulate normal life activities by pumping antibiotics, QS signal molecules, and virulence factors out of the cell [59]. When the efflux pump MexCD-OprJ was mutated, the HHQ output of *P. aeruginosa* decreased significantly [60]. In addition, the efflux pump MexEF-OprN was found to play a role in outputting HHQ [61]. Therefore, we speculated that PQS is also secreted into the extracellular space through an unknown efflux pump pathway. Recently, it was reported that the MacB transporter (MacB is part of the MacA-MacB-TolC efflux pump) encoded by PA4063-4066 may be involved in PCH secretion in *P. aeruginosa* [62]. This work shows that the MacB transporter can be used as a protective mechanism against cobalt (Co) toxicity [62]. During this process, excess intracellular Co may form a complex with PCH. The PCH–Co complex is then pumped out by the MacB exporter [62] (Figure 2). Given that both PCH and PQS are hydrophobic and share cellular entry pathways [6,9,12,16,63], we speculate that both PCH and PQS may be transported to the extracellular space through the MacB exporter.

## 4. Regulatory Correlation

**Figure 3:**
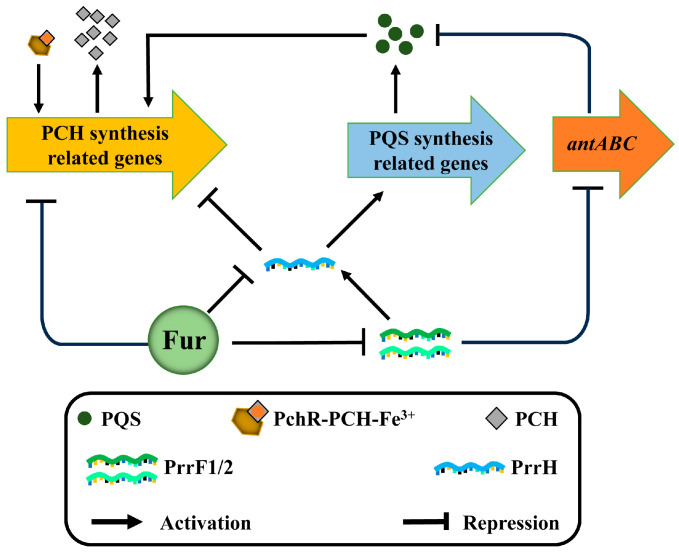
Schematic representation of the PCH and PQS regulatory correlation. (1) Fur binds to the promoter regions of PCH and sRNA PrrF1, PrrF2, and PrrH to directly inhibit the synthesis of PCH and sRNA. In addition, PrrH inhibits the expression of PCH synthesis genes and promotes the expression of PQS synthesis genes. PrrF1/2 promotes the production of PQS by inhibiting the expression of the anthranilic acid (substrate of PQS synthesis) degradation gene, *antABC*. Therefore, Fur also indirectly regulates the biosynthesis of PCH and PQS by inhibiting the transcription of the sRNA PrrF1/2 and PrrH. (2) PQS induces the expression of PCH synthesis-related genes.

Ferric uptake regulator (Fur) can regulate the biosynthesis of PCH and PQS simultaneously [6,64] (Figure 3). This mode of regulation can be divided into direct and indirect. Under iron-rich conditions, Fur can form homodimers with Fe^2+^ and bind to specific sequences in the promoter region of the target gene to inhibit the expression of the target gene [3,6]. For instance, Fur directly represses PCH biosynthesis by binding to the promoters of *pchDCBA* and *pchEFGHI* under iron-rich conditions [5,65]. In addition, Fur can indirectly regulate the synthesis of PCH by inhibiting the transcription of the sRNA PrrF1, PrrF2, and PrrH [66,67,68,69]. PrrF1 and PrrF2 are arranged in tandem on the genome of *P. aeruginosa* [66], and the two can form the third sRNA, PrrH, together with the sequence of the spacer region [66,70]. PrrH complements the mRNA sequence of the PCH synthesis gene *pchE*, which inhibits its expression and ultimately inhibits the synthesis of PCH [66]. Unlike PCH, Fur regulates the synthesis of PQS solely through sRNA [66,67,68,69]. When *P. aeruginosa* is under iron-limiting conditions, PrrF1/2 inhibit the *antABC* gene for the degradation of anthranilate (substrate of PQS synthesis) to promote PQS production [71]. Furthermore, PrrH appears to promote the synthesis of PQS [66]. It has been shown that the expression of the PQS biosynthetic proteins PqsB, PqsC, and PqsD in *P. aeruginosa* decreased after PrrH deletion [66]. In short, Fur, as the core regulator of iron homeostasis, can regulate the synthesis of PCH and PQS in a variety of ways to help *P. aeruginosa* adapt to changing environments.

In addition to being regulated by Fur, PQS can induce the expression of PCH synthesis-related genes [54,72,73]. When 40 μM of PQS is added to wild-type *P. aeruginosa*, the expression of PCH synthesis genes *pchA*, *pchB*, *pchC*, *pchD*, *pchE*, *pchM*, and *pchG* is significantly upregulated [72]. To further confirm this result, thin-layer chromatography (TLC) analysis was used to monitor the production of PCH in the PQS-supplemented cultures [72]. The results show that the addition of PQS increases the production of PCH in *P. aeruginosa*, and this phenomenon can be reversed by adding excess iron [72]. The main reason for this result is the iron starvation response of *P. aeruginosa* caused by iron chelation of PQS [72]. Furthermore, when *pqsA* or *pqsE* is deleted, the expression of genes involved in the synthesis, uptake, and regulation of PCH in *P. aeruginosa* (*pchA*, *pchB*, *pchD*, *pchE*, *pchF*, *pchI*, *pchR*, and *fptA*) is significantly reduced [54,73]. These results indicate that PQS can induce the expression of PCH synthesis-related genes (Figure 3).

## 5. Conclusions

*P. aeruginosa* is listed by the World Health Organization as one of the pathogens in urgent need of the development of new antibiotics [74]. It causes infection by overcoming the host immune response [16]. To achieve this purpose, *P. aeruginosa* secretes many virulence factors, such as siderophore PCH [75], responds to environmental stress, and regulates infection activity through the *pqs* QS system [76]. Previous studies only focused on the individual functions of PCH or PQS and did not consider whether there was a synergistic effect between them [12,56]. This review updates the biosynthetic process of PCH and PQS and discusses the relationship between PCH and PQS from the following aspects: (1) PCH and PQS have iron chelating characteristics and can share one outer membrane transporter (FptA) and three inner membrane transporters (FptX, PchHI, and FepBCDG) to mediate iron uptake. (2) Fur simultaneously regulates the biosynthesis of PCH and PQS through the transcription of the sRNA PrrF1, PrrF2, and PrrH. (3) PQS can induce the expression of PCH synthesis-related genes. Based on this, we speculate that PCH and PQS may be related in more aspects. First, since PCH and PQS share transporters mediating iron uptake [9,16], there may be functional synergy between them. Second, since PCH and PQS share the same uptake pathway [9,16], they may share the same secretory pathway. Third, since both PCH and PQS can chelate other metal ions in addition to iron ions [52,77], they may be jointly involved in the uptake of other metal ions. Finally, since both PCH and PQS can be used as signaling molecules to regulate their own synthesis [6,76], they may influence each other’s signaling effects. Considering the above points, it is necessary to further explore and verify the functional correlation between PCH and PQS, which will greatly improve the current understanding of the adaptation of *P. aeruginosa* to complex environments and provide a special perspective for the prevention and treatment of *P. aeruginosa* infections.

## Data Availability

Not applicable.
